# Tumor treating induced fields: a new treatment option for patients with glioblastoma

**DOI:** 10.3389/fneur.2024.1413236

**Published:** 2024-10-17

**Authors:** Zehao Cai, Zukai Yang, Ying Wang, Ye Li, Hong Zhao, Hanwen Zhao, Xue Yang, Can Wang, Tengteng Meng, Xiao Tong, Hao Zheng, Zhaoyong He, Chunli Niu, Junzhi Yang, Feng Chen, Zhi Yang, Zhige Zou, Wenbin Li

**Affiliations:** ^1^Department of Neuro-oncology Cancer Center, Beijing Tiantan Hospital, Capital Medical University, Beijing, China; ^2^School of Basic Medical Science, Capital Medical University, Beijing, China; ^3^Kunlun Tripot (Beijing) Medical Technology Co., Ltd., Beijing, China; ^4^School of Integrated Circuits, Huazhong University of Science and Technology, Wuhan, Hubei, China; ^5^School of Biomedical Engineering, Capital Medical University, Beijing, China

**Keywords:** electromagnetic therapy, glioblastoma, central nervous system, electromagnetic induction, transformer

## Abstract

**Purpose:**

Currently, a range of electromagnetic therapies, including magnetic field therapy, micro-currents therapy, and tumor treating fields, are under investigation for their potential in central nervous system tumor research. Each of these electromagnetic therapies possesses distinct effects and limitations. Our focus is on overcoming these limitations by developing a novel electric field generator. This generator operates by producing alternating induced currents within the tumor area through electromagnetic induction.

**Methods:**

Finite element analysis was employed to calculate the distribution of electric fields. Cell viability was assessed using the CCK-8 assay. Tumor volumes and weights served as indicators to evaluate the effectiveness of TTIF. The *in-vivo* imaging system was utilized to confirm tumor growth in the brains of mice.

**Results:**

TTIF significantly inhibited the proliferation of U87 cells both *in vitro* and *in vivo*.

**Conclusion:**

TTIF significantly inhibited the proliferation of U87 cells both *in vitro* and *in vivo*. Consequently, TTIF emerges as a potential treatment option for patients with progressive or metastatic GBM.

## Introduction

The dysregulation of biological characteristics in tumors arises from changes occurring at both the cellular and tissue levels. One mechanism of dysregulation is through bioelectrical changes (1). Tumor cells exhibit a resting membrane potential of approximately −25 mV, significantly lower than that of normal cells (2). Moreover, multiple ion channels are found to be overexpressed in various types of tumor cells (3–6). Consequently, tumor cells disrupt local ionic environments, resulting in the generation of distinct local electric fields (EFs) (7). These EFs are present within the tumor interior and on its surface, leading to outward electric currents at tumor sites (8). The differences in metabolism, structure, and electrical properties between tumors and normal tissues provide the mechanistic basis for electromagnetic therapy to selectively kill tumor cells through non-thermal effects, while minimally impacting normal cells.

Low-frequency (<100 Hz) alternating magnetic fields (MFs) and pulsed magnetic fields generated by the coil exhibit anti-tumor effects by inducing cell apoptosis, oxidative stress, increasing intracellular calcium levels, and reducing angiogenesis (9–15). One direct effect of magnetic field therapy is the disruption of ion movement by the Lorentz force. Another hypothesis suggests that alternating magnetic fields induce currents within tumors. Research on central nervous system (CNS) tumors has indicated that MFs enhance the apoptotic effects of temozolomide (TMZ) through redox regulation in U87 cells (16, 17). However, there is a lack of relevant clinical-level studies.

Common current therapies include direct current therapy (DCT) and alternating current therapy. DCT involves inserting electrodes into tumors and delivering stable direct current (40–80 mA) at low voltage (6–8 V). Direct current exerts its anti-tumor effects through electrochemical reactions, anti-angiogenesis, and altering the pH of the surrounding environment (18–20). One study demonstrated that sustained exposure to low-frequency (50 Hz), low-intensity (7.5 μA) alternating current can impact the proliferation of rat glioma C6 cells, and increasing the frequency and intensity can enhance its cytotoxic effect (21). Additionally, alternating current with a frequency of 100–200 kHz, intensity of 10–50 mA, and intermittent exposure (30 min/day) significantly inhibits the proliferation of breast cancer cells and glioma cells (22). However, due to the requirement of surgical implantation, there is currently a lack of clinical research on CNS tumors.

Tumor treating fields (TTFields) delivered by a pair of insulated electrodes are an intermediate-frequency (100–300 kHz), low-intensity(1–3 V/cm), alternating electric fields (23, 24). The early proposed TTFields’ anti-tumor mechanism of action involved polymerization-depolymerization process of microtubules and mitotic disruption interfered by electrical forces on cell structure proteins (25). Recent research showed that TTFields can exert anti-tumor effects through multiple mechanisms, including disrupting cell membrane potential, increasing cell membrane permeability, affecting calcium ion channels, damaging DNA and inhibiting DNA repair (26–29). Currently, multiple clinical trial results demonstrated that TTFields have excellent anti-tumor effects in various types of cancers, including glioblastoma (GBM), malignant pleural mesothelioma (MPM), non-small cell lung cancer (NSCLC), and pancreatic carcinoma (PAC) (30–38). The median overall survival (OS) time of patients with newly diagnosed glioblastoma received temozolomide-only is 16.0 months. When TTFields are administrated, the median OS time is 20.9 months. Due to the unique treatment form of TTFields, it can not only treat tumors alone but is also particularly suitable for combination with other treatment methods, such as radiotherapy (RT), chemotherapy, targeted therapy, and immunotherapy (39). TTFields therapy has demonstrated promising results in the treatment of GBM when combined with targeted therapies such as bevacizumab. And one case report described a patient with thalamic glioblastoma who achieved a complete radiological response following treatment with proton therapy, temozolomide (TMZ), and TTFields (40). Multiple combination therapies incorporating TTFields are currently in Phase 2 clinical trials.

Over the past 20 years, numerous preclinical studies on electromagnetic therapy for CNS tumors have shown promising results, but clinical studies have been very limited. The unique tissue structure and biological functions of the CNS have posed barriers to the translation of devices into clinical practice. The application of invasive electromagnetic devices has been approached with caution. Even the TTFields device, which is a capacitor-like device delivering electric fields, has limitations. Insulated electrodes are placed on the shaved scalp when patients receive TTFields therapy. While the existing TTFields device has demonstrated efficacy against supratentorial GBM, its efficacy against infratentorial and spinal cord GBM has not been confirmed (41). It is challenging to arrange two opposite arrays on the face and the skin adjacent to the spinal cord to ensure that the threshold of electric field intensity is sufficient to arrest cellular proliferation (42).

We are committed to addressing these limitations by developing a new electric field generator. We have found that a transformer-like electric fields device offers several advantages, including the feasibility of vertical electric fields covering the infratentorial and spinal cord areas, wearability, and non-disposable packaging. The device generates alternating induced currents in the tumor area based on electromagnetic induction. In the present study, we propose and validate, for the first time to our knowledge, the feasibility of Tumor-treating Induced Fields (TTIF) therapy delivered by a transformer-like electric fields device.

## Methods

### TTIF device

**Figure 1:**
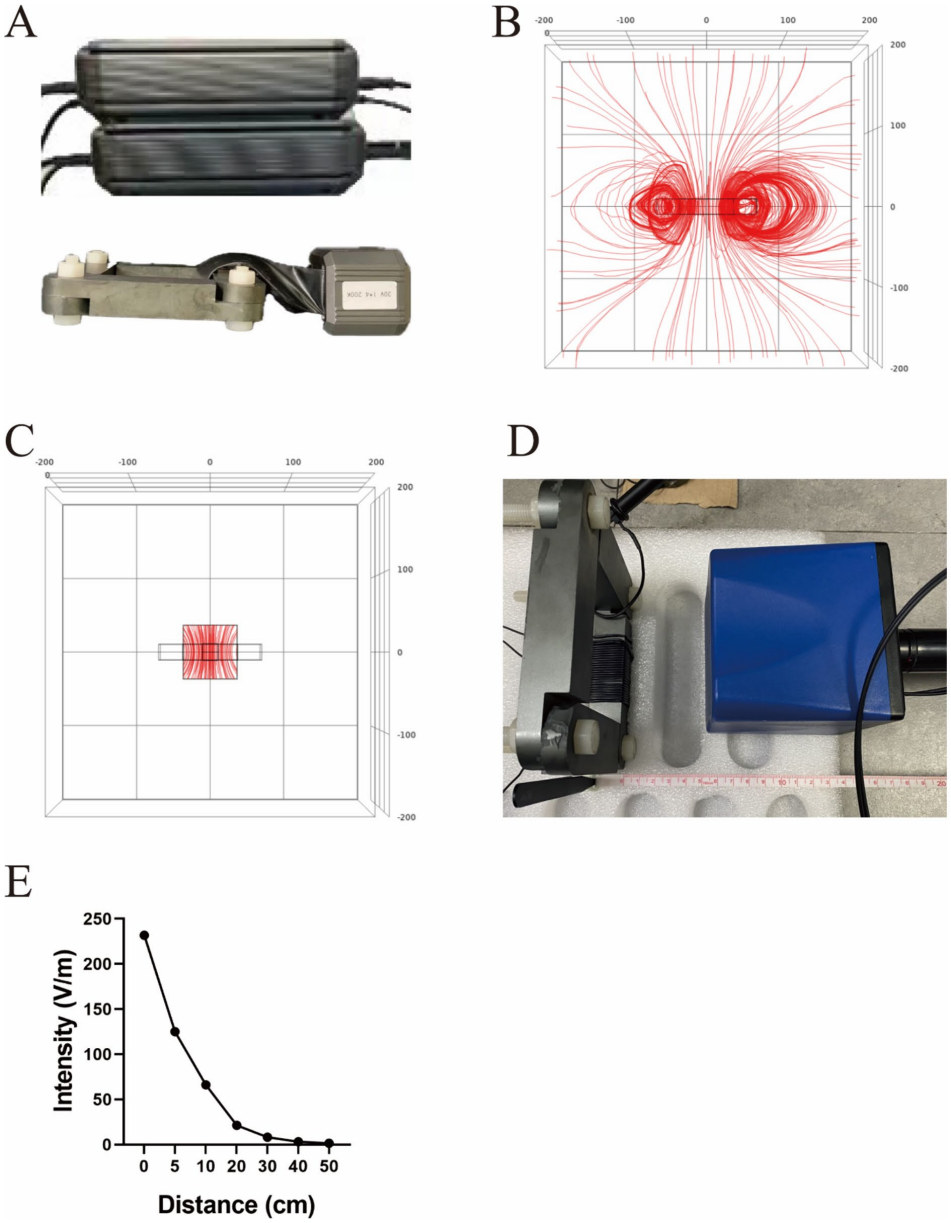
Structure diagram of the TTIF device **(A)**. Distribution of electric field lines generated by TTIF **(B)**. Vertical electric field lines at the center of the magnetic ring **(C)**. Detection of electric field intensity **(D)**. Relationship between electric field intensity and distance **(E)**.

The TTIF device mainly consists of one motor, wires, one capacitor, and one magnetic ring (Figure 1A). The electric coil wound around the magnetic core, together with the capacitor, forms an LC resonance circuit. The switch on the LC resonance circuit is turned off after the motor is powered once, and energy is continuously transferred in the inductor and capacitor. Based on electromagnetic induction, alternating current in the inductor coil generates an alternating magnetic field within the magnetic ring. Then, the alternating magnetic field within the magnetic ring generates an alternating electric field radiating outward. Consequently, the tumor microenvironment exhibits micro-alternating induced currents. The function of the switch and the LC resonance circuit is to convert the low voltage direct current in the wire into high voltage, medium-frequency alternating current in the inductor coil. In cellular experiments, the current density in the tumor cell region reached 1,000 mA/m^2^. This result was obtained through finite element analysis.

### Finite element analysis

The electric field distribution around the device was calculated using the finite element method to solve the quasi-static approximation of the Maxwell’s equations, which is valid for this model. For the model we utilized the Comsol Multiphysics, version 6.2. The following boundary conditions were imposed: continuity of the normal component of the current density at all interior boundaries and electric insulation at the external boundaries. The frequency was set to 200 kHz.

### Cell viability

In this study, TTIF was applied to glioblastoma cells at 200 kHz, based on previous research. The cell dish was positioned at the center of the magnetic ring, perpendicular to its plane. Cell viability was assessed using the Cell Counting Kit-8 (CCK-8). A total of 1 × 10^5 cells were seeded into a 35 mm culture dish and incubated overnight. At each time point, the medium was replaced with 1 mL of media containing 10% CCK-8 reagent and incubated for 1–2 h at 37°C with 5% CO_2_. Subsequently, the media from each 35 mm culture dish were transferred into 96-well plates (100 μL/well). The absorbance of each well was measured at 450 nm using a microplate reader.

### Animal models

Both subcutaneous and intracranial xenograft tumor models were utilized to evaluate the effect of TTIF on GBM *in vivo*.

### The subcutaneous tumor models

Female BALB/c-nu mice aged 6 weeks were obtained from Beijing Si Bei Fu Experimental Animal Technology Co., Ltd. Subcutaneous injections of U87 GBM tissue (8mm^3^) with 200 μL phosphate-buffered saline (PBS) were administered in the right groin of the mice. Successful inductions of 75 mm^3^ subcutaneous tumors were observed within 10 days. The mice were randomly divided into different groups: Control or Tumor-Treating Induced Fields (TTIF) groups. The maximum allowable tumor size in the mice before euthanasia was 2,000 mm^3^. Tumors were isolated and measured at the end of the experiment. Tumor volumes were calculated using the following formula: width^2 × length × 0.52.

### The brain tumor models

A total of 0.32 μL of the G261-luc cell suspension was injected into the brains of C57BL/6 mice, approximately 1.8 mm lateral and 1 mm posterior to the bregma in the right brain hemisphere, over 4 min using a stereotactic rodent brain injection system. In total, either 1 × 10^4 or 1 × 10^5 G261-luc glioma cells were injected. Mice underwent bioluminescence imaging with an *in-vivo* imaging system (IVIS) before and after treatment to confirm tumor growth. Total flux (p/s) was calculated from the Region of Interest (ROI) in Living Image Software to quantitatively assess treatment efficacy.

### Statistical analysis

Statistical analyses were conducted using GraphPad Prism 8.0.1. One-way ANOVA tests were utilized to compare tumor volumes and total flux between treatment groups. The normality of data was assessed using the Shapiro–Wilk test. Unpaired *t*-tests were employed to compare tumor weight and cell viability. Log-rank tests were conducted to compare overall survival (OS) between two groups. A *p*-value of <0.05 was considered statistically significant. Numerical values were reported as mean ± standard error of mean (SEM). When P is greater than or equal to 0.05, the figure is labeled with “ns.” When P is less than 0.05 but greater than or equal to 0.01, the figure is labeled with “*.” When P is less than 0.01, the figure is labeled with “**.”

## Results

### The TTIF device generated a vertical electric field at the center of the magnetic ring

Initially, a quadrilateral magnetic ring was utilized as the electric field generator, and FEA was conducted to analyze the electric field distribution. The results indicated that the TTIF device produced circular, closed electric fields surrounding the magnetic ring (Figure 1B). As proximity to the center of the magnetic ring increased, the curvature of the electric field lines decreased, tending towards perpendicularity to the plane of the magnetic ring (Figure 1C). Electric field intensity in the air surrounding the magnetic ring was measured (Figure 1D), showing values exceeding 50 V/m within a 10 cm range (Figure 1E).

### TTIF inhibited the proliferation of U87 cells *in vitro*

**Figure 2:**
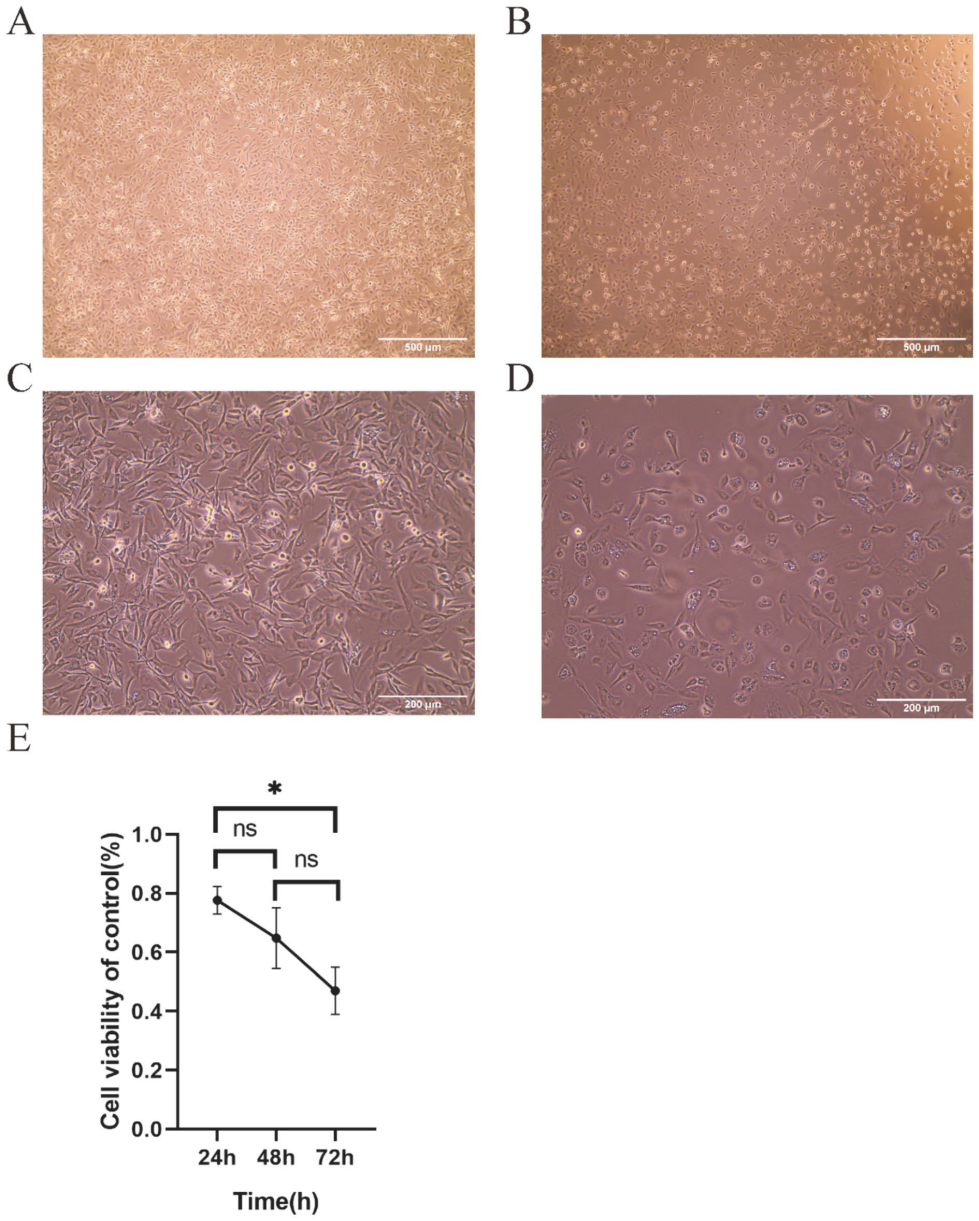
The 30 V TTIF device can inhibit the proliferation of U87 cells, achieving a current density of up to 1,000 mA/m^2^ in the cell area. Comparison of cell images between the control group **(A)** and the electric field group **(B)** under a 4x phase-contrast microscope. Cell images of the control group **(C)** and the electric field group **(D)** under a 10x phase-contrast microscope. Relationship between cell viability and TTIF exposure time **(E)**.

Following 72 h of TTIF treatment with a current density exceeding 1,000 mA/m^2^, U87 cell density markedly decreased, accompanied by noticeable alterations in cell morphology (Figures 2A,B). Circular cell proportion increased, while cytoplasmic vacuoles emerged (Figures 2C,D). The inhibitory effect of TTIF was found to be dependent on exposure time, with efficacy increasing with prolonged treatment durations (Figure 2E).

### TTIF inhibited the growth of GBMs in the subcutaneous murine model

**Figure 3:**
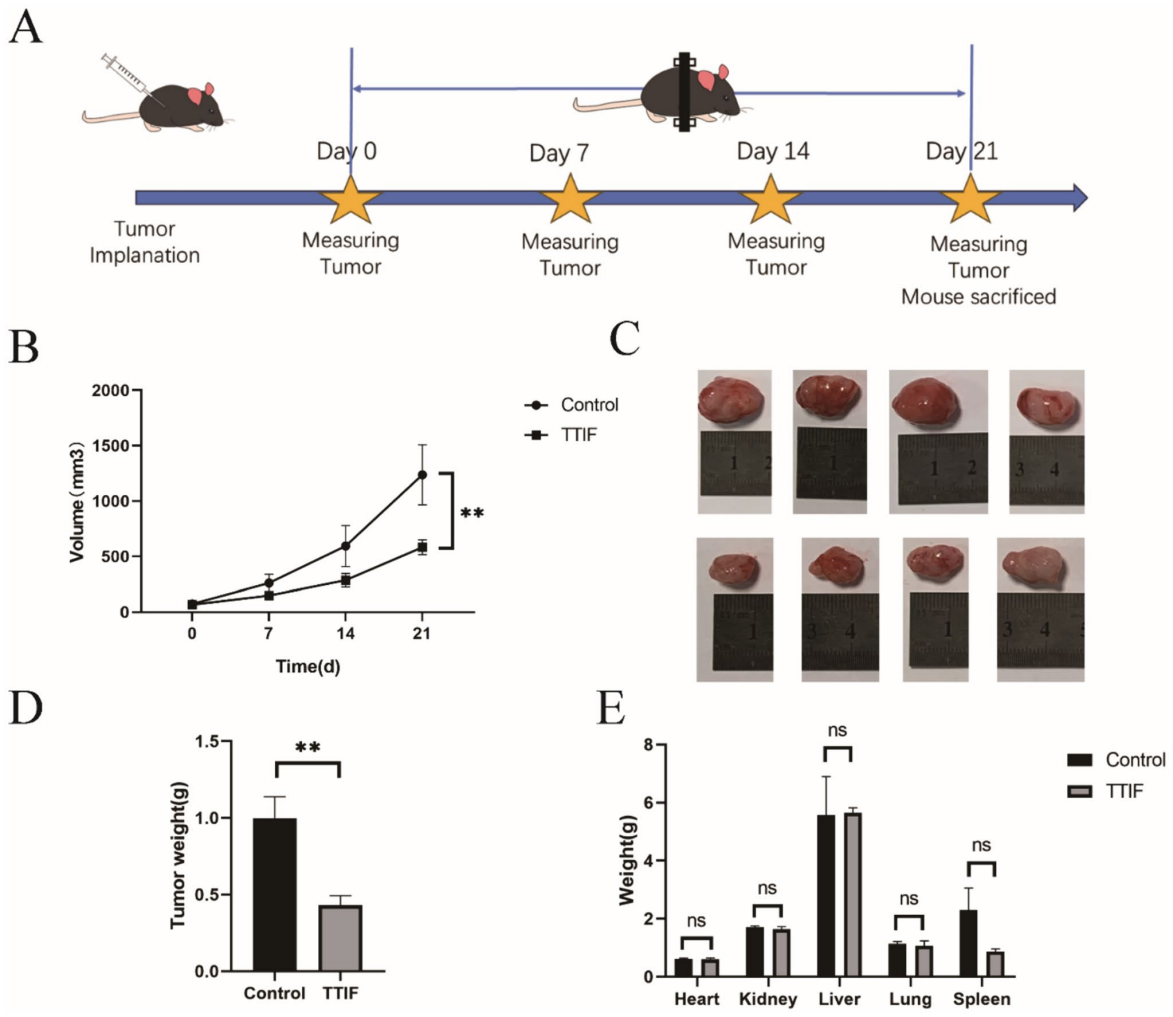
BALB/c-nu mice were chosen for the experiment involving subcutaneous tumor formation, and the voltage of the TTIF device was set to 30 V. Schedule of TTIF treatment for subcutaneous tumor-bearing mice **(A)**. Relationship between tumor volume and TTIF treatment duration **(B)**. Comparison of tumor sizes between the control group mice and the TTIF group mice **(C)**. Comparison of tumor weights between the control group mice and the TTIF group mice **(D)**. Comparison of organ weights between the control group mice and the TTIF group mice **(E)**.

To investigate the anti-tumor effects of TTIF *in vivo*, we initially transplanted U87 tissue subcutaneously into BALB/c-nu mice (*n* = 4 for each group). The tumor-bearing mice in the TTIF group were housed at the center of the magnetic ring and received continuous TTIF treatment for 21 days. Tumor volume was assessed every 7 days using a caliper (Figure 3A). The time-tumor volume curve indicated that TTIF significantly suppressed the growth of subcutaneous glioma volumes in mice (*p* = 0.007, Figure 3B). Following 21 days of TTIF treatment, the tumor volume of the experimental group mice was notably smaller than that of the control group mice (Figure 3C). Supporting this observation, the data on tumor weight also demonstrated a significant difference (*p* = 0.010, Figure 3D). Additionally, we assessed the weight of various organs in mice, with results showing no statistically significant difference between the two groups (Figure 3E).

### TTIF prolonged the OS of intracranial tumor-bearing mice

**Figure 4:**
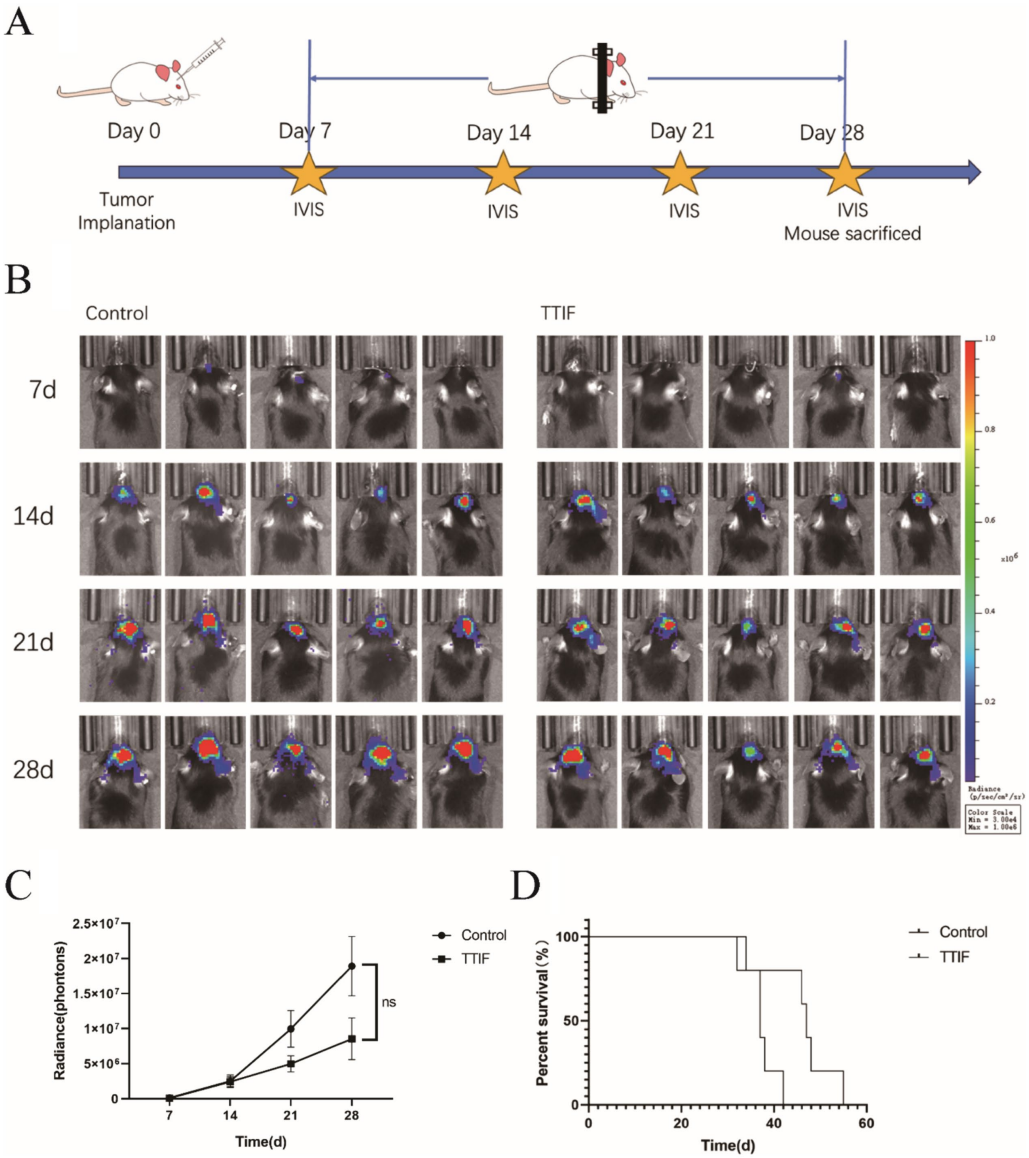
BALB/c-nu mice were chosen for the experiment involving intracranial tumor formation, and the voltage of the TTIF device was set to 45 V. Schedule of TTIF treatment for intracranial tumor mice **(A)**. Bioluminescence imaging’s of tumors at various time points **(B)**. Relationship between tumor fluorescence intensity and time **(C)**. Comparison of OS between the TTIF group mice and the control group mice **(D)**.

1 × 10^4^ G261 glioma cells were injected into the brains of C57 mice (*n* = 10), and IVIS was used on days 7, 14, 21, and 28 (Figure 4A). On day 7, after confirming successful induction of brain tumors using IVIS, mice were randomly divided into control and TTIF groups. Although the difference was not statistically significant, we observed a trend of decreasing luciferase intensity in mice receiving TTIF treatment compared to the control group (*p* = 0.0826, Figures 4B,C).

To further investigate TTIF’s ability to inhibit tumor growth in the *in situ* brain tumor murine model, the number of cells injected was increased to 1 × 10^5^. On day 3, mice were randomly divided into control and TTIF groups. Subsequently, we recorded the OS of each mouse. TTIF-treated mice showed prolonged survival, with a median survival of 47 days compared to 37 days in the control group (*p* = 0.0274, Figure 4D).

### The characteristics of the small magnetic ring

**Figure 5:**
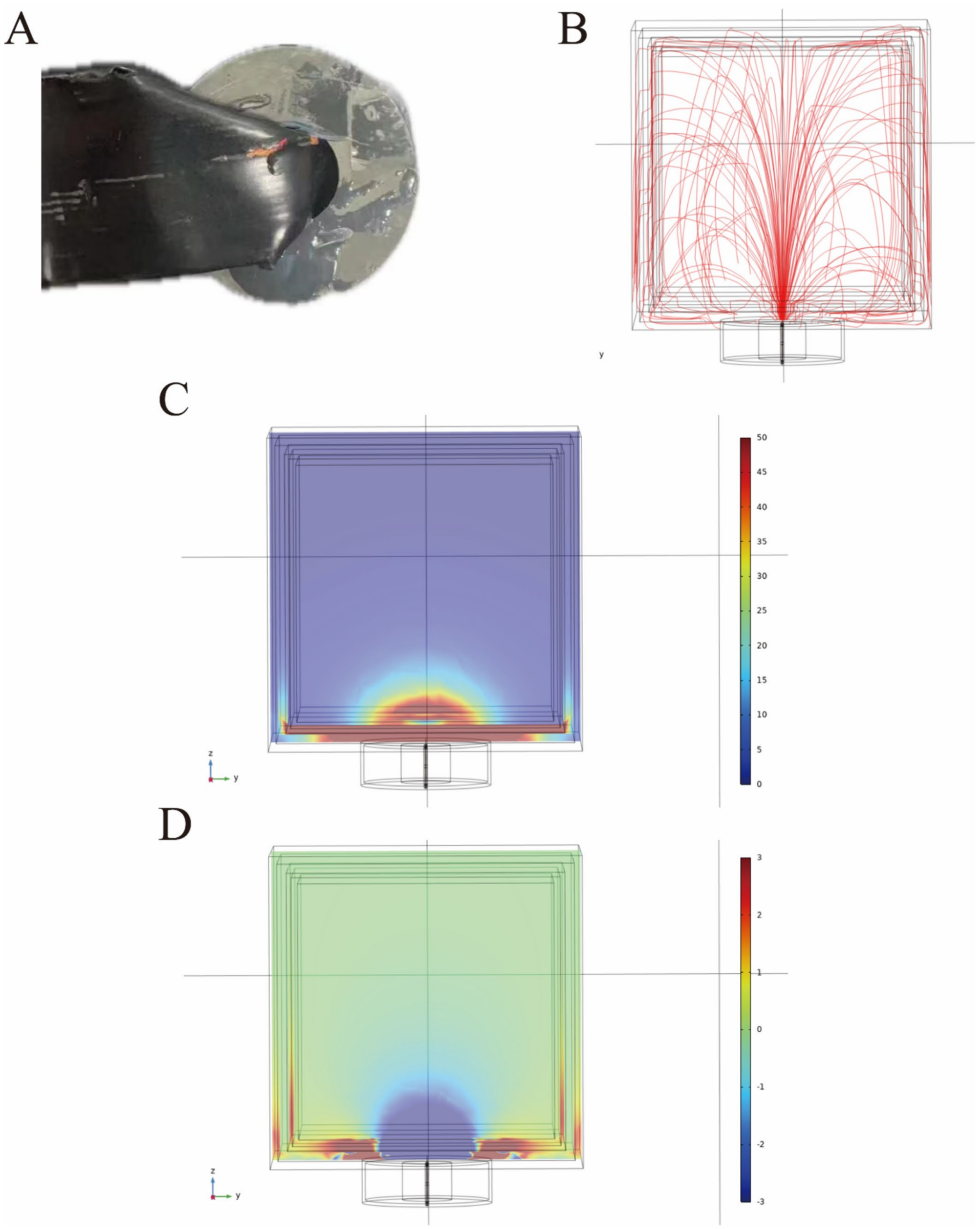
The appearance of the small magnetic ring **(A)**. The small magnetic ring generates a radial, geyser-like distribution of electric field lines in the head **(B)**. The distribution of the electric field in human head tissue under a voltage of 120 V is expressed in volts per meter (V/m) **(C)**. Additionally, the distribution of induced longitudinal conduction current in the human head is presented, with current density expressed in amperes per square meter (A/m^2^) **(D)**.

In previous research, we thoroughly examined the characteristics and verified the therapeutic efficacy of a large magnetic ring. Subsequently, we pursued the development of a smaller magnetic ring, measuring 4 cm in external diameter and 1.6 cm in internal diameter (Figure 5A). However, we encountered challenges stemming from inadequate miniaturization and insufficient reduction in weight of the smaller ring, impeding its applicability in animal experiments involving tumor-bearing mice. To overcome this hurdle, we devised a simplified cubic model of human head tissue for finite element analysis, aimed at investigating the behavior of small magnetic coils. This model comprehensively incorporates the scalp, skull, cerebrospinal fluid, gray matter, and white matter, each with distinct thicknesses of 5 mm, 6 mm, 3 mm, and 4 mm, respectively. Upon situating the small magnetic ring on the surface of the head tissue, a radial fountain-like distribution of electric field lines manifests within the head (Figure 5B). Upon reaching voltage levels comparable to those of clinical TTFields equipment, we observed the emergence of a specific intensity of electric field and longitudinal induced conduction current in the vicinity of the brain, adjacent to the ring (Figures 5C,D).

## Discussion

GBM stands as the most aggressive primary tumor affecting the central nervous system (43). The standard treatment protocol for newly diagnosed GBM involves surgery followed by radiotherapy (RT) concurrently with TMZ, along with adjuvant TMZ, optionally supplemented with TTFields (44). Advanced stages of glioblastoma exhibit notably aggressive characteristics (45). Approximately 4.5% of patients diagnosed with supratentorial glioblastoma experience infratentorial metastases, while 3–5% present with metastatic spinal dissemination (MSD) (46, 47). Autopsy findings have revealed frequent incidental spread from supratentorial regions to the brain stem and spine, in contrast to relatively infrequent clinical incidences (48, 49). Complications such as infratentorial recurrence (ITR) and MSD may occur more frequently. Presently, there exists no standardized treatment approach for managing ITR and MSD. Although these patients may undergo additional radiotherapy and chemotherapy, their median OS, which are 5.5 months for ITR and 4 months for MSD, significantly lag behind those of the general GBM patient population (9.1 months).

The grim prognosis observed in GBM patients is partly attributed to the challenges associated with successful drug delivery across the blood–brain barrier (BBB) (50). The presence of the BBB limits the availability of traditional chemotherapy and targeted drugs for GBM. Since 2005, only a few new drugs—namely, Temozolomide, bevacizumab, and regorafenib—have been included in the NCCN guidelines as first- and second-line treatments for glioblastoma GBM (51, 52). However, research into new treatments for GBM is advancing rapidly (53). One promising option is vemurafenib, a highly selective BRAF V600 inhibitor that has demonstrated long-term antitumor effects in some patients with BRAF V600 mutant gliomas (54). Additionally, combination therapy targeting both BRAF and MEK has shown advantages over monotherapy with BRAF inhibitors. In a study involving the combination of dabrafenib and trametinib for recurrent or refractory high-grade gliomas (HGG) with the BRAF V600E mutation, an objective response was observed in 32% of GBM patients, with a complete response in 6.5% of cases (55). Furthermore, paxalisib, a small molecule capable of penetrating the blood–brain barrier and inhibiting the PI3K/AKT/mTOR pathway, has demonstrated clinical activity in newly diagnosed GBM patients with unmethylated MGMT promoters (56).

During radiation therapy, particularly reirradiation, the tolerance of normal brain tissue to radiation doses emerges as a significant limiting factor (54). Another important factor in qualifying patients for re-radiation is the increased risk of radionecrosis. The two primary directions in the development of radiotherapy for central nervous system tumors are: (1) modifying the radiotherapy regimen, including approaches such as preoperative radiotherapy and phased radiotherapy; and (2) enhancing the capabilities of radiotherapy equipment, exemplified by advancements in gamma knife and proton therapy technologies (57).

Electromagnetic therapy presents itself as a potentially viable option for treating CNS tumors. However, when utilizing TTFields, the range of EFs remains highly restricted. While TTFields delivered through capacitor-like devices demonstrate effectiveness primarily for supratentorial GBM, their application may not extend to infratentorial and spinal cord GBM. Consequently, patients with GBM face a dearth of sufficient treatment options when tumors progress or metastasize.

TTIF emerges as a potential treatment option for these patients. The TTIF device generates an alternating electric field at the center and on both sides of the magnetic ring through a circular alternating magnetic field. When tissues or tumors are in proximity to the TTIF device, alternating currents are induced. The device is non-invasive and easy to wear. The small magnetic ring is positioned on the skin surface corresponding to the tumor’s location. Compared to TTFields electrodes, the advantage of TTIF’s small magnetic ring is that it can be used individually, allowing placement on the skin atop the head or over the cerebellum. With a larger magnetic ring, tumors experience vertical induced currents at the center of the magnetic ring.

TTIF can be utilized clinically in various forms. When used alone as an alternative to TTFields, TTIF effectively treats tumors located within a large magnetic ring placed over the body, such as the head, as well as those within a specific range above and below the plane of the ring. Additionally, a small magnetic ring can be worn similarly to a transcranial magnetic stimulation (TNS) therapy device, generating a radial TTIF to treat tumors throughout the body. TTIF offers comparable and enhanced benefits when combined with other treatments. There is ongoing debate regarding the potential impact of wearing a TTFields device on the efficacy of radiation therapy. The necessity to remove TTFields can also lead to increased treatment costs due to the disposable nature of the electrodes. In contrast, TTIF equipment is designed for easy wear and removal, providing added convenience. Furthermore, TTIF can complement the effects of TTFields therapy. When TTFields are employed to treat supratentorial tumors, TTIF can be utilized as an adjunct therapy to prevent supratentorial metastases or to address spinal-disseminated tumors. Further FEA is required to determine specific treatment options for both scenarios.

Our study is subject to several limitations. The frequency and induced current density utilized in cellular experiments with the TTIF device were derived from various prior studies. In our initial study, we focused exclusively on 200 kHz, which is recognized as the most sensitive frequency for TTFields treatment of GBM cells. However, it is important to note that the electric field characteristics of TTIF may differ from those of TTFields. These differences could include variations such as non-conserved electric fields and conservative electric fields, potentially resulting in distinct efficacy and frequency sensitivity between the two treatments. However, due to the design of the LC resonance circuit, which causes these two physical parameters to vary together, the relationship between frequency and current density and their effective threshold was not established in this study. Furthermore, the efficacy of the small magnetic ring has not been validated in animal experiments, primarily because the ring has not been adequately miniaturized to reduce weight. Additionally, further research is warranted to elucidate additional mechanisms of action.

## Conclusion

We introduced the transformer-like induced fields/currents device for the first time in the field of electromagnetic therapy, outlining its feasible device structure and testing its functionality. Our findings indicate that TTIF significantly inhibited the proliferation of U87 cells both *in vitro* and *in vivo*. Consequently, TTIF emerges as a potential treatment option for patients with progressive or metastatic GBM.

## Data Availability

The raw data supporting the conclusions of this article will be made available by the authors, without undue reservation.
